# Case of Vesico-Vaginal Fistula and Spontaneous Cure

**Published:** 1866-08

**Authors:** Lorenzo Hubbard

**Affiliations:** Marysville, California


*Case of Vesico-Vaginal Fistula and Spontaneous Cure.* By LORENZO HUBBARD, M. D., Marysville, California.

Mrs. F.——, of Yuba county, suffered instrumental labor, Nov. 3, 1865. From the commencement to the termination of labor was some five or six hours. Presentation natural—vertex to the left acetabulum. The membranes ruptured early, and the head soon passed the superior strait and became impactad in the pelvis. After many ineffectual attempts to bring the head forward, the forceps were applied and the child delivered without any extraordinary effort. No untoward symptoms followed.

At the end of three days the patient was able to sit up to have her bed changed, and at the end of two weeks was sufficiently recovered to sit up most of the day, and to walk about the house. At this time, and after taking a dose of senna, she was attacked with what she supposed were griping pains, from the medicine; but the pains were so severe and continued so long that she finally sent for me to visit her. I found her suffering as above; pains bearing down and occurring at irregular and distant intervals. The bowels had been moved moderately by the medicine, and I very naturally supposed the womb was acting through sympathy. I accordingly prescribed anodyne, and promised to see her the next day.

In the morning I was informed that after a paroxysm of severe bearing down pain, some time in the night, there had been an involuntary discharge of water, and from that time she had no control over the urinary organs; and, moreover, she had discharged a yellowish concretion resembling very much tartar which frequently collects on teeth.

An examination being necessary, I passed two fingers up the vagina, and soon met with what I supposed to be a rent through its walls. After a more thorough manipulation I was satisfied that the fingers passed into the bladder; the calculus concretions appearing abundant and quite firmly adherent to that viscus.

I then inserted a glass speculum, and was astonished and alarmed to discover the entire destruction of a portion of the urethra and an open lesion extending above the neck of the bladder. The urine trickled down through the opening and instrument continually, while portions of the whitish incrustations which seemed to cover the entire cavity, passed away. I made an unsuccessful attempt to scoop out the calculi from the bladder; the adhesions, however, were so firm that it was deemed prudent to adopt a milder method. With this view injections of warm water, acidulated with sulphuric acid, were used, and succeeded perfectly in the course of a couple of days.

When a sound was passed into the bladder through the urethra, a space of about two inches on the sound could be seen by means of the speculum in the vagina.

After the concretions had been discharged the parts appeared fresh and healthy. No pain and very little soreness were experienced. Immediately, healthy granulations began to sprout from the superior portion of the cavity extending downwards, and soon very nearly covered the abraded parts, hiding from view the fistula, which at first easily admitted the passage of two fingers.

The cause of the difficulty is enveloped in some doubt. Where the parts bruised in delivery, and was the destruction the result of ulceration? In this case, would there not have been pain, inflammation, and at least some irritative fever? Or may it not have been the result of absorption, occasioned by the pressure of calculous matter, and possibly somewhat assisted by the counter pressure of the child's head? It is evident that no lesion took place at the time of delivery, as no inconvenience was experienced until two weeks had elapsed.

The parts having been thoroughly cleansed, as was before stated, a solution of sulphate of zinc was ordered to be used as an injection, morning and evening, and occasionally, when dark patches appeared, the nitrate of silver in substance was applied, in the hope of favoring healthy granulations.

During the first month the urine dripped away continually, as it was secreted by the kidneys. At the end of the second month, there appeared a slight disposition of the bladder to retain its contents, and the patient was delighted to experience for the first time, since the accident, something like a desire to urinate.

After the expiration of three months, there was very little dripping when the patient was in a recumbent or sitting posture, or when she was standing or walking, except when the bladder was partially filled. On the first of March examination proved that a portion, at least, of the urine escaped through the meatus urinarius without the assistance of the catheter.

At the present time, April 10th, the patient is able to walk or stand for a considerable time without much inconvenience; if on her feet most of the day she would perhaps wet two napkins. There is no involuntary escape of urine when sitting or lying. The desire to urinate is natural, and the bladder possesses its normal expulsive powers. The fistula cannot, as formerly, be discovered through the speculum, nor can we feel the metallic sound; nevertheless there is still an escape of urine into the vagina.

From the neck of the bladder, at the superior extremity of the injury, a heavy mass of granulations, as before mentioned, sprouted, extending in the course of the urethra, completely covering the "fistula." This mass is perhaps two and a half inches in length and one and a half in breadth, resembling the section of an egg with the small end downwards. Underneath this point, until the last examination, I have been able to pass an instrument into the bladder; but at the last attempt the patient complained so much that I desisted, under the pleasing prospect that the fistula had nearly closed. The catheter is easily passed through the urethra into the bladder, although the orifice at the neck appears somewhat contracted, the patient complaining of a disagreeable distention when the instrument is inserted.

The sequel of this case is only accumulative evidence that nature, left to herself, or led by a kindly hand, is capable of astonishing reparative efforts; and teaches the instructive lesson, that, however far removed from probable human aid the accident may appear, the unfortunate sufferer may still trust with reasonable confidence to her powers.

This case has been a novelty, it being the first I had ever witnessed. "Mr. Barnes, of Exeter, England," some years ago reported two cases. (See Dr. Gooch's Compendium of Midwifery, page 55.) These were supposed to have been produced by the continuous pressure of the child's head during a protracted labor. Dr. Gooch was consulted, and recommended the "introduction into the vagina of an India rubber bottle with a piece of sponge sewed to it;" the sponge was placed opposite the fistulous opening in the bladder, which absorbed the urine and conducted it into the bottle. A catheter was passed two or three times a day. Here the urethra was perfect. In the case we have reported, the urethra was severed. At the end of two months, in Mr. Barnes' cases, the "fistulous communication was much smaller." "And in a few months the opening was entirely closed."

To relieve this dripping of urine, I used for a time, a sponge and bladder; but this soon proved inconvenient, and the patient dropped its use altogether, preferring napkins properly adjusted; and, moreover, I became convinced that the plugging was a disadvantage.

First. It distends the parts and necessarily creates much irritation, and prevents the natural and rapid contraction, so essential in the cure.

Second. It invites morbid secretions, and the parts cannot be kept in so perfect a state of cleanliness and quietude as when only injections are relied upon.

While we trust mostly to the efforts of nature for the cure, if one is effected, the surgeon should be vigilant in seeing that the parts are kept scrupulously clean; that ulcerations, if they occur, are promptly checked, and that the bladder be kept as free from the accumulation of urine as possible by means of the catheter.

